# Rapid identification of inflammatory arthritis and associated adverse events following immune checkpoint therapy: a machine learning approach

**DOI:** 10.3389/fimmu.2024.1331959

**Published:** 2024-03-15

**Authors:** Steven D. Tran, Jean Lin, Carlos Galvez, Luke V. Rasmussen, Jennifer Pacheco, Giovanni M. Perottino, Kian J. Rahbari, Charles D. Miller, Jordan D. John, Jonathan Theros, Kelly Vogel, Patrick V. Dinh, Sara Malik, Umar Ramzan, Kyle Tegtmeyer, Nisha Mohindra, Jodi L. Johnson, Yuan Luo, Abel Kho, Jeffrey Sosman, Theresa L. Walunas

**Affiliations:** ^1^ Center for Health Information Partnerships, Northwestern University Feinberg School of Medicine, Chicago, IL, United States; ^2^ Feinberg School of Medicine, Northwestern University, Chicago, IL, United States; ^3^ Department of Medicine, Division of Rheumatology, Northwestern University Feinberg School of Medicine, Chicago, IL, United States; ^4^ Hematology and Oncology, University of Illinois Health, Chicago, IL, United States; ^5^ Department of Preventive Medicine, Northwestern University Feinberg School of Medicine, Chicago, IL, United States; ^6^ Center for Genetic Medicine, Northwestern University Feinberg School of Medicine, Chicago, IL, United States; ^7^ Department of Medicine, Division of Oncology, Northwestern University Feinberg School of Medicine, Chicago, IL, United States; ^8^ Robert H. Lurie Comprehensive Cancer Center of Northwestern University, Chicago, IL, United States; ^9^ Departments of Pathology and Dermatology, Northwestern University Feinberg School of Medicine, Chicago, IL, United States; ^10^ Department of Medicine, Division of General Internal Medicine, Northwestern University Feinberg School of Medicine, Chicago, IL, United States

**Keywords:** immune checkpoint inhibitors, immune-related adverse events, immune checkpoint inhibitor-induced inflammatory arthritis, machine learning, electronic health records, big data

## Abstract

**Introduction:**

Immune checkpoint inhibitor-induced inflammatory arthritis (ICI-IA) poses a major clinical challenge to ICI therapy for cancer, with 13% of cases halting ICI therapy and ICI-IA being difficult to identify for timely referral to a rheumatologist. The objective of this study was to rapidly identify ICI-IA patients in clinical data and assess associated immune-related adverse events (irAEs) and risk factors.

**Methods:**

We conducted a retrospective study of the electronic health records (EHRs) of 89 patients who developed ICI-IA out of 2451 cancer patients who received ICI therapy at Northwestern University between March 2011 to January 2021. Logistic regression and random forest machine learning models were trained on all EHR diagnoses, labs, medications, and procedures to identify ICI-IA patients and EHR codes indicating ICI-IA. Multivariate logistic regression was then used to test associations between ICI-IA and cancer type, ICI regimen, and comorbid irAEs.

**Results:**

Logistic regression and random forest models identified ICI-IA patients with accuracies of 0.79 and 0.80, respectively. Key EHR features from the random forest model included ICI-IA relevant features (joint pain, steroid prescription, rheumatoid factor tests) and features suggesting comorbid irAEs (thyroid function tests, pruritus, triamcinolone prescription). Compared to 871 adjudicated ICI patients who did not develop arthritis, ICI-IA patients had higher odds of developing cutaneous (odds ratio [OR]=2.66; 95% Confidence Interval [CI] 1.63-4.35), endocrine (OR=2.09; 95% CI 1.15-3.80), or gastrointestinal (OR=2.88; 95% CI 1.76-4.72) irAEs adjusting for demographics, cancer type, and ICI regimen. Melanoma (OR=1.99; 95% CI 1.08-3.65) and renal cell carcinoma (OR=2.03; 95% CI 1.06-3.84) patients were more likely to develop ICI-IA compared to lung cancer patients. Patients on nivolumab+ipilimumab were more likely to develop ICI-IA compared to patients on pembrolizumab (OR=1.86; 95% CI 1.01-3.43).

**Discussion:**

Our machine learning models rapidly identified patients with ICI-IA in EHR data and elucidated clinical features indicative of comorbid irAEs. Patients with ICI-IA were significantly more likely to also develop cutaneous, endocrine, and gastrointestinal irAEs during their clinical course compared to ICI therapy patients without ICI-IA.

## 1 Introduction

Immune checkpoint inhibitors (ICIs) have become a pillar of cancer therapy, with demonstrated efficacy in many malignancies (1–9). ICIs are antibodies that antagonize checkpoints of T-cell development, enabling tumor-reactive T-cells to attack cancer cells (1, 2, 8, 10, 11). There are currently two major classes of ICIs. The first, approved in 2011, targets cytotoxic T-lymphocyte antigen 4 (CTLA-4). The second, first approved in 2014, targets programmed cell death protein 1 (PD-1) or its counterpart, programmed cell death ligand 1 (PD-L1). These ICIs may be used alone or combined with each other and other cancer therapies and are approved for a wide panel of cancers. An estimated 44% of US cancer patients are eligible for ICI therapy (4–7, 9, 12, 13).

However, while ICIs are effective anti-cancer agents, checkpoint blockade is associated with development of immune-related adverse events (irAEs) that affect a wide spectrum of organ systems (14–17). IrAEs can pose a significant barrier to ICI usage. They can prevent patients from continuing ICIs, diminish patient quality of life, and in severe cases, lead to death (3, 12, 13, 18). There are two major deficits in our current understanding of irAEs and our ability to care for patients with irAEs. First, irAEs are difficult to identify both in the patient care setting and for research studies. Outside of large clinical trials, many published studies are single-site with small cohorts identified and characterized by labor-intensive chart review (19–24). Second, we currently have a very limited ability to predict which patients will develop irAEs. Rheumatologic irAEs, including ICI-induced inflammatory arthritis (ICI-IA), epitomizes both of these limitations (24–35). ICI-IA has been recognized as an irAE for less than a decade with case studies and small cohort descriptions first appearing in the literature in 2017 (24–26). With a reported prevalence of 1-7%, it is relatively rare (36). However, it can have significant impact for patients with 12-13% of cases resulting in termination of ICI therapy (34, 35). Previous studies have described ICI-IA affecting the knees or small joints, tending to be seronegative for anti-cyclic citrullinated peptide (CCP) antibodies and rheumatoid factor, and most commonly treated with corticosteroids with elevation to disease-modifying antirheumatic drugs (DMARDs), tumor necrosis factor (TNF) inhibitors, and interleukin (IL)-6 receptor inhibitors (24–27, 29, 32, 37). Though development of ICI-IA cannot be well predicted, in a previous study, melanoma and genitourinary cancer as well as receiving combination ICI therapy were found to be associated with ICI-IA development compared to lung cancer and PD-1 monotherapy, respectively (38).

Prompt identification of patients with ICI-IA is essential for providing rapid referrals for effective clinical care and for performing research into the etiology of ICI-IA. However, ICI-IA is difficult to identify in clinical data, owing to its rarity, lack of dedicated diagnosis code, and heterogeneous presentation (24–32, 35). Furthermore, ICI-IA’s typically lower severity compared to other irAE such as myocarditis or pneumonitis means that patients may be less likely to be seen by a rheumatologist for their symptoms. While there have been numerous studies published on ICI-IA, ICI-IA cohort definition has primarily relied on manual identification of these patients by rheumatologists and/or filtering for patients who have been seen by a rheumatologist or been prescribed specific immunomodulatory drugs (26, 27, 29, 32, 34, 38). As a result, cohorts have remained relatively small and single site and run the risk of missing many ICI-IA patients who may have had a less severe presentation or other barriers to seeing a rheumatologist. Additionally, while many studies on ICI-IA include description of comorbid irAEs (26–28, 32), no previous study has tested the association of ICI-IA with these comorbid irAEs compared to a control cohort of ICI patients who did not develop arthritis, making it difficult to understand if the prevalence of comorbid irAEs experienced by ICI-IA patients is statistically different from the rest of the ICI population.

To address these fundamental gaps in knowledge and facilitate identification of ICI-IA and associations with the development of ICI-IA, we developed an electronic health record (EHR)-based machine learning strategy to rapidly identify patients who have possible ICI-IA and discover hidden features associated with ICI-IA. With the wide adoption of EHRs in inpatient and ambulatory settings (39), EHR data and data modeling strategies present an opportunity to develop tools to identify ICI-IA, as well as uncover clinical features of ICI-IA or associated conditions that may not be immediately obvious. Many previous studies have demonstrated success in identifying conditions including hypertension, stroke, systemic lupus erythematosus, asthma, and leukemia (40–44). To investigate the relationship between ICI-IA and other irAEs elucidated by our machine learning model, we also fully adjudicated a control cohort of 871 patients receiving ICI therapy for all irAEs, and used this control cohort to examine the association between the development of ICI-IA and other comorbid irAEs as well as cancer type and ICI regimen.

## 2 Materials and methods

### 2.1 Patient population and data source

The study population included patients seen in the Robert H. Lurie Comprehensive Cancer Center at Northwestern Medicine (NM), a large healthcare system providing inpatient, outpatient, and specialty care throughout Chicago and Northern Illinois. Data was acquired from the Northwestern Medicine Enterprise Data Warehouse (NMEDW), NM’s clinical research database containing data on over 10 million patients as of October 2023. This study was governed by the Northwestern University institutional review board, protocol #STU00210502 and STU00206779.

**Figure 1:**
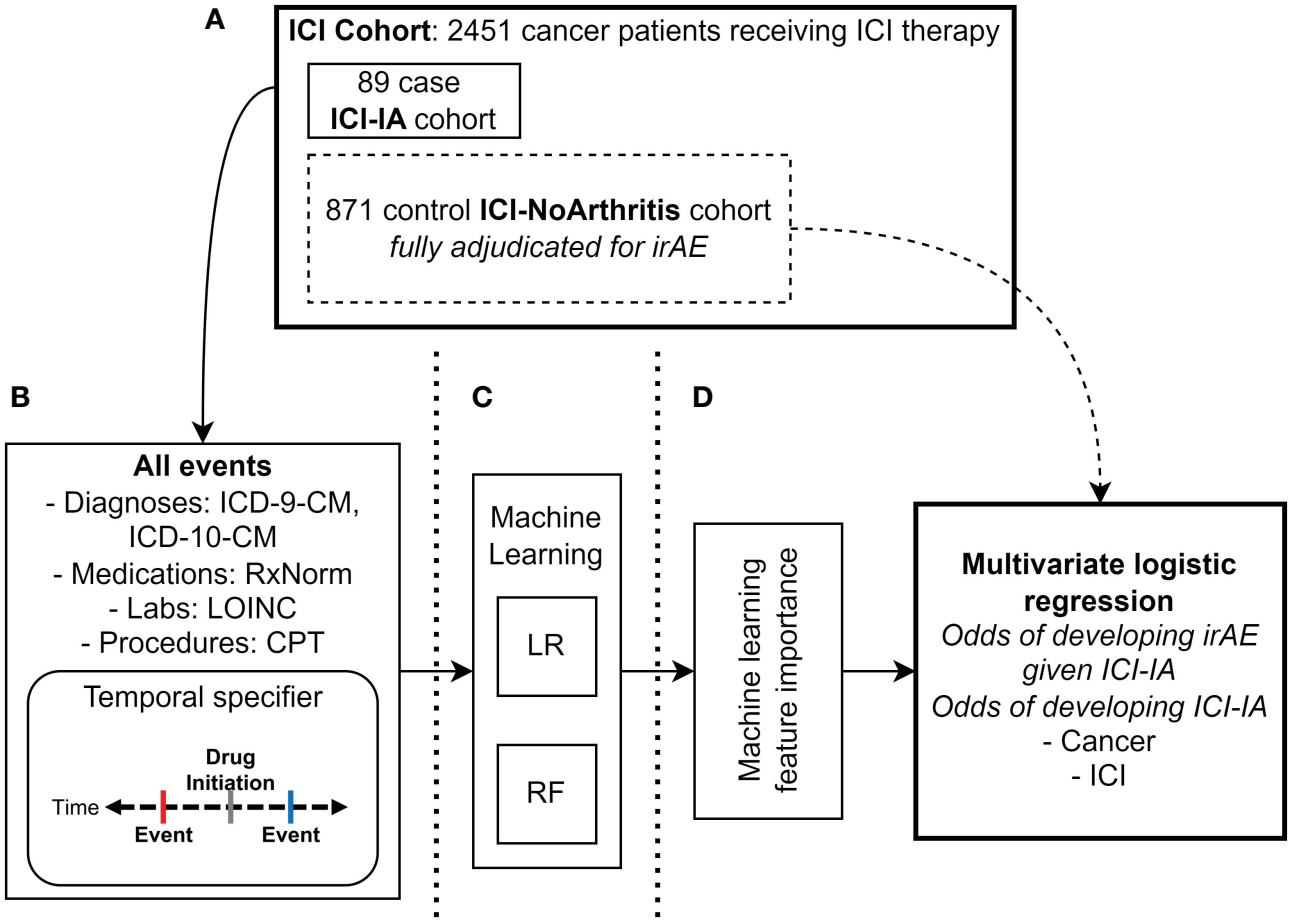
Methods diagram. **(A)** Manual adjudication of ICI cohort (N=2451 patients) for immune checkpoint inhibitor-induced inflammatory arthritis (ICI-IA) cases (N=89 patients). From the ICI cohort, 871 random patients without ICI-IA (ICI-NoArthritis) were adjudicated for all irAE. **(B)** Electronic health record data extraction of all diagnosis (ICD-9-CM, ICD-10-CM), medication (RxNorm), laboratory test (LOINC), and procedure (CPT) codes. Individual code occurrences were modified to specify whether they occurred before or after ICI initiation, and dichotomized to presence/absence of the code. Data was extracted for the full ICI cohort of 2451 patients. **(C)** Logistic regression (LR) and random forest (RF) machine learning models were trained on the EHR codes to identify ICI-IA. **(D)** Feature importance was analyzed to characterize ICI-IA patients in the EHR. Multivariate logistic regression was used to calculate odds ratios for development of ICI-IA given cancer and ICI regimen, as well as development of non-arthritis irAEs given ICI-IA versus ICI-noIA.

We retrospectively identified an ICI cohort of all patients aged 18 to 99 with a diagnosis of cancer (melanoma, renal cell carcinoma, non-small cell and small cell lung carcinoma, urothelial cancer, head and neck cancer, gastric cancer, colon cancer, liver cancer, cervical cancer, uterine cancer, breast cancer, Hodgkin’s lymphoma, Merkel cell carcinoma, rectal cancer, prostate cancer, esophageal cancer, leukemia, or lymphoma) who received at least one dose of ICI therapy (pembrolizumab, nivolumab, cemiplimab, atezolizumab, avelumab, durvalumab, ipilimumab, or tremilumumab) between March 1, 2011 and January 1, 2021 (**Figure 1A**). Cancer was identified in the NMEDW by International Classification of Disease-9^th^ revision-Clinical Modification (ICD-9-CM) and ICD-10-CM diagnosis codes. ICIs were identified in the NMEDW by a regular expression search for the generic and brand name in the medication data table (**Supplementary Table 1**). Sex, race, and ethnicity were gathered from patient demographic data present in the NMEDW. Prior autoimmune diseases were collected from NMEDW diagnosis codes (**Supplementary Table 2**).

### 2.2 Adjudication of ICI-induced inflammatory arthritis and statistical test control cohorts

Patient charts in NM’s Epic EHR were manually reviewed (SDT) with clinician guidance (CG, JL, AK, JS) to identify a case cohort of patients with ICI-IA (**Figure 1A**). We reviewed the charts of all patients who had the keywords: “arthritis”, “arthralgia”, or “joint” in the assessment and plan section of any clinical notes written after the patient received their first ICI dose. Cases were classified with ICI-IA if the patient had *de novo* joint pain/arthralgia/arthritis (no history of arthritis or presentation different from what the patient has experienced in the past), and an oncologist or rheumatologist noted suspicion of the presentation being secondary to ICI therapy. Case status, date of ICI-IA onset, cancer, ICI regimen, joint involvement, rheumatoid factor, anti-CCP, anti-nuclear antibodies (ANA), and treatment for ICI-IA were recorded in a REDCap database (45). Cases were additionally reviewed for other irAEs the patients experienced – cutaneous AEs, thyroid dysfunction, hypophysitis or adrenal insufficiency, diabetes, hepatic AEs, diarrhea, colitis, pneumonitis, cardiovascular AEs, and encephalitis. They were classified with an irAE if an oncologist or the relevant specialist noted suspicion of the presentation being secondary to ICI therapy without other likely etiologies. See **Supplementary Methods** for details.

The remaining patients without ICI-IA in the full ICI cohort were used as controls for our machine learning models. To compare irAE associations with ICI-IA, a random sample of 871 patients without ICI-IA (ICI-NoArthritis) from the overall ICI cohort of 2451 were chart reviewed for all irAEs (same irAEs and classification threshold as above) to serve as the control cohort for the statistical tests (SDT, GMP, KJR, CDM, JDJ, JT, KV, PD, SM, UR) (**Figure 1A**). Following initial adjudication, SDT reviewed a random sample of 10% of the charts and found greater than 90% agreement.

### 2.3 EHR code selection for machine learning

We collected all EHR clinical codes for every patient in our ICI cohort: ICD-9-CM and ICD-10-CM diagnosis codes, Logical Observation Identifiers Names and Codes (LOINC) laboratory codes, Unified Medical Language System (UMLS) RxNorm medication codes, and Current Procedural Terminology (CPT) procedure codes (**Figure 1B**). To determine if a machine learning model could select sensible ICI-IA-relevant codes and discover new predictors of ICI-IA, we input all EHR codes into our models. ICD-9-CM diagnosis codes were translated to ICD-10-CM to prevent duplication of diagnosis codes, using the concept relationships in the Observational Medical Outcomes Partnership (OMOP) common data model vocabulary tables (46). For each code, we added a temporal modifier specifying whether the code occurred before or after ICI initiation. We then dichotomized each to presence or absence of the code.

### 2.4 Machine learning to identify ICI-induced inflammatory arthritis in EHR data

The EHR codes were fed into Logistic Regression with Ridge (L2) penalty and Random Forest machine learning models to classify patients who experienced ICI-IA from the ICI cohort (**Figure 1C**). These models were selected for their capacity to provide clinically interpretable models. We bootstrapped model development 100 times to calculate model performance metrics with 95% confidence intervals and consensus code contribution. For each round of model development (each 1 of 100 bootstrap rounds), we used a random 50/50 training-testing split, stratified for consistent case/control proportions in the training and test sets. Cases were up-sampled during cross-validation and model training to balance the low ratio of ICI-IA cases to ICI controls (47). Five-fold cross-validation with 25 iterations was used to optimize model parameters in the training set. The test set was left untouched for performance evaluation. Models were evaluated using area under the receiver operating characteristic curve (AUROC). Accuracy, sensitivity, specificity, positive predictive value (PPV), and negative predictive value (NPV) were measured for all models, optimizing Youden’s J to balance sensitivity and specificity (48). EHR code contribution was calculated from the logistic regression beta coefficients and the random forest feature importance.

### 2.5 Key EHR codes in the ICI-induced inflammatory arthritis machine learning model

Feature importance from the random forest model was analyzed for the key EHR codes used to identify ICI-IA, averaging feature importance across the 100 bootstrapped models – codes with higher feature importance contribute more to the model for identifying ICI-IA (**Figure 1D**). While not a direct equivalence, these codes could indicate clinical features potentially describing ICI-IA and allow us to determine if the models were using ICI-IA-relevant information to capture patients with ICI-IA. The Fisher Exact test was used to determine the association between ICI-IA and the codes. Models were trained on decreasing percentages of the top codes (50% - 0.003%) and performance compared to the full models.

### 2.6 Cancer type and immune checkpoint inhibitor association with ICI-induced inflammatory arthritis

Multivariate logistic regression was used to calculate unadjusted and adjusted odds ratios (ORs) of developing ICI-IA given cancer type and first ICI regimen (specific ICI drug) (**Figure 1D**). Lung cancer and pembrolizumab were used as reference groups for cancer type and ICI regimen, respectively, as they represented our largest cancer type and treatment regimen. Covariates included in the calculation of adjusted ORs were sex, age, race, and ethnicity, cancer type for ICI ORs and ICI regimen for cancer ORs. Cancer type was determined by ICD-9/10 codes and ICI was determined by regex search as described in the patient population section (**Supplementary Table 1**). Cancer determination by ICD code compared to chart review showed <10% discrepancy. An ICI regimen was determined combination ICI therapy if two different ICIs were infused on the same date.

### 2.7 Determining irAE associations with ICI-induced inflammatory arthritis

Multivariate logistic regression was used to calculate unadjusted and adjusted ORs of developing non-arthritis irAEs given ICI-IA, compared to ICI-NoArthritis control patients (**Figure 1D**). IrAE associations were tested at three timeframes relative to ICI-IA development: irAE development any time after ICI initiation, only irAEs occurring prior to ICI-IA development, and only irAEs occurring post ICI-IA development. In the ICI-NoArthritis controls, irAE were included at any time after ICI initiation. Covariates included in the calculation of adjusted ORs were sex, age, race, ethnicity, cancer type, first ICI regimen, and presence of autoimmune disease prior to ICI initiation.

### 2.8 Analytical software

Statistical analysis and machine learning was performed using R 4.2.2 and Python 3.9.7 with *scikit-learn*, *imbalanced-learn*, *scipy*, and *statsmodels* packages. Statistical test results were considered statistically significant where p < 0.05. Models are available on GitHub (https://github.com/stevetran99/ICI-IA_ML_Classification).

## 3 Results

### 3.1 ICI-induced inflammatory arthritis cohort clinical characteristics

From the NMEDW, 2451 patients with a diagnosis of cancer who received at least one ICI dose between March 1, 2011 and January 1, 2021 were identified. Expert-guided chart review identified 89 cases of ICI-IA based on clinical suspicion without other likely etiologies in the oncology and rheumatology clinical notes. There were 2362 patients without ICI-IA remaining from the ICI cohort to serve as machine learning controls, with 871 of those patients adjudicated for other irAE serving as controls for our association tests.

**Table 1** presents the demographic and cancer characteristics of the ICI-IA cohort and machine learning control cohort. We saw no significant differences across primary demographic characteristics (sex, age, race, ethnicity). Lung cancer (N=34, 38%), melanoma (N=29, 33%), and kidney cancer (N=20, 23%) were the most common cancers in our ICI-IA cohort. Pembrolizumab (N=28, 32%), nivolumab (N=23, 26%), and combination nivolumab-ipilimumab (N=27, 30%) were the predominant first ICI regimens and the majority of patients had no prior autoimmune disease (N=87, 98%). ICI-IA involved the knees (N=42, 47%), hand (N=31, 35%) and shoulder (N=28, 31%) (**Table 2**). The knees (N=30, 34%) and hands (N=22, 25%) were the most common first joints affected. Twenty-seven patients had diffuse joint pain. Of patients with specific joint involvement mentioned (N=71, 80%), the median number of distinct joint locations affected was 2, range 1 to 7. Of those tested for markers of rheumatologic disease, 5 of 20 patients were positive for rheumatoid factor, 0 of 16 patients were positive for anti-CCP antibodies, 9 of 15 patients were positive for ANA (**Table 2**). Eighteen (20%) patients were seen by a rheumatologist. ICI-IA onset was a median of 20 weeks (IQR 8-45 weeks) after initiating checkpoint therapy. Primary treatment modalities for the ICI-IA symptoms were steroids (N=43, 48%), and NSAIDs (N=33, 37%). In 18 (20%) patients, ICI therapy was held or stopped entirely due to arthritis.

**Table 1:** Demographic and clinical variables for cancer patients receiving Immune Checkpoint Inhibitor Therapy between March 1, 2011 and January 1, 2021.

	Overall	ICI-IA	ML Control
	N=2451	N=89	N=2362
Sex, n (%)			
Male	1355 (55.3)	48 (53.9)	1307 (55.3)
Female	1096 (44.7)	41 (46.1)	1055 (44.7)
Age, n (%)			
20 – 29	10 (0.4)		10 (0.4)
30 – 39	43 (1.8)	1 (1.1)	42 (1.8)
40 – 49	101 >(4.1)	5 (5.6)	96 (4.1)
50 – 59	284 (11.6)	15 (16.9)	269 (11.4)
60 – 69	716 (29.2)	29 (32.6)	687 (29.1)
70 – 79	776 (31.7)	25 (28.1)	751 (31.8)
80 – 89	441 (18.0)	13 (14.6)	428 (18.1)
>= 90	80 (3.3)	1 (1.1)	79 (3.3)
Race, n (%)			
White	1935 (78.9)	77 (86.5)	1858 (78.7)
Black or African American	198 (8.1)	4 (4.5)	194 (8.2)
Asian	96 (3.9)	1 (1.1)	95 (4.0)
Other	111 (4.5)	5 (5.6)	106 (4.5)
Unknown	111 (4.5)	2 (2.2)	109 (4.6)
Ethnicity, n (%)			
Hispanic or Latino	109 (4.4)	2 (2.2)	107 (4.5)
Not Hispanic or Latino	2220 (90.6)	84 (94.4)	2136 (90.4)
Unknown	122 (5.0)	3 (3.4)	119 (5.0)
Cancer, n (%)			
Lung Cancer	1278 (52.1)	34 (38.2)	1244 (52.7)
Melanoma	495 (20.2)	29 (32.6)	466 (19.7)
Renal Cell Carcinoma	323 (13.2)	20 (22.5)	303 (12.8)
Breast Cancer	23 (0.9)	1 (1.1)	22 (0.9)
Urothelial Cancer	143 (5.8)	2 (2.2)	141 (6.0)
Endometrial Cancer	13 (0.5)	1 (1.1)	12 (0.5)
Other Malignancy	57 (2.3)	2 (2.2)	55 (2.3)
Cervical Cancer	1 (0.0)		1 (0.0)
Colon Cancer	17 (0.7)		17 (0.7)
Esophageal Cancer	4 (0.2)		4 (0.2)
Gastric Cancer	4 (0.2)		4 (0.2)
Head and Neck Cancer	52 (2.1)		52 (2.2)
Hodgkins Lymphoma	1 (0.0)		1 (0.0)
Leukemia	6 (0.2)		6 (0.3)
Liver Cancer	6 (0.2)		6 (0.3)
Merkel Cell Carcinoma	6 (0.2)		6 (0.3)
Other Lymphoma	6 (0.2)		6 (0.3)
Prostate Cancer	15 (0.6)		15 (0.6)
Rectal Cancer	1 (0.0)		1 (0.0)
First ICI, n (%)			
Pembrolizumab	941 (38.4)	28 (31.5)	913 (38.7)
Nivolumab	635 (25.9)	23 (25.8)	612 (25.9)
Ipilimumab	119 (4.9)	3 (3.4)	116 (4.9)
Combination*	332 (13.5)	27 (30.3)	305 (12.9)
Durvalumab	147 (6.0)	5 (5.6)	142 (6.0)
Atezolizumab	271 (11.1)	3 (3.4)	268 (11.3)
Avelumab	5 (0.2)		5 (0.2)
Cemiplimab	1 (0.0)		1 (0.0)
Prior Autoimmune Disease, n (%)			
No Prior	2339 (95.4)	87 (97.8)	2252 (95.3)
Prior	112 (4.6)	2 (2.2)	110 (4.7)

ICI-IA, Immune checkpoint inhibitor-induced inflammatory arthritis; ML, Machine learning.

*Combination: Nivolumab-Ipilimumab.

**Table 2:** Immune checkpoint inhibitor-induced inflammatory arthritis (ICI-IA) laboratory tests, joint involvement, and comorbid irAEs for our cohort of patients with ICI-IA.

	ICI-IA
	N=89
Rheumatoid factor, n (%)	
Positive	5 (6)
Negative	15 (17)
Anti-CCP Antibodies, n (%)	
Positive	0 (0)
Negative	16 (18)
Anti-Nuclear Antibodies, n (%)	
Positive	9 (10)
Negative	6 (7)
Arthritis Treatment, n (%)	
Steroids	43 (48)
NSAID	33 (37)
Joint Injection	9 (10)
DMARD	6 (7)
Apremilast	1 (1)
None Documented	31 (35)
Joint Involvement, n (%)	
Hand	31 (35)
Wrist	12 (13)
Elbow	5 (6)
Shoulder	28 (31)
Neck	5 (6)
Back	9 (10)
Hip	18 (20)
Knee	42 (47)
Ankle	13 (15)
Foot	7 (8)
Diffuse	27 (30)
Comorbid irAE, n (%)	
Cutaneous	38 (43)
Endocrine	
Thyroid	16 (18)
Hypophysitis/Adrenal Insufficiency	10 (11)
Diabetes	1 (1)
Gastrointestinal	
Diarrhea/Constipation	26 (29)
Colitis	9 (10)
Hepatic	8 (9)
Pneumonitis	4 (4)
Cardiac	1 (1)
Encephalitis	1 (1)

### 3.2 EHR-based machine learning reliably identified ICI-induced inflammatory arthritis

**Figure 2:**
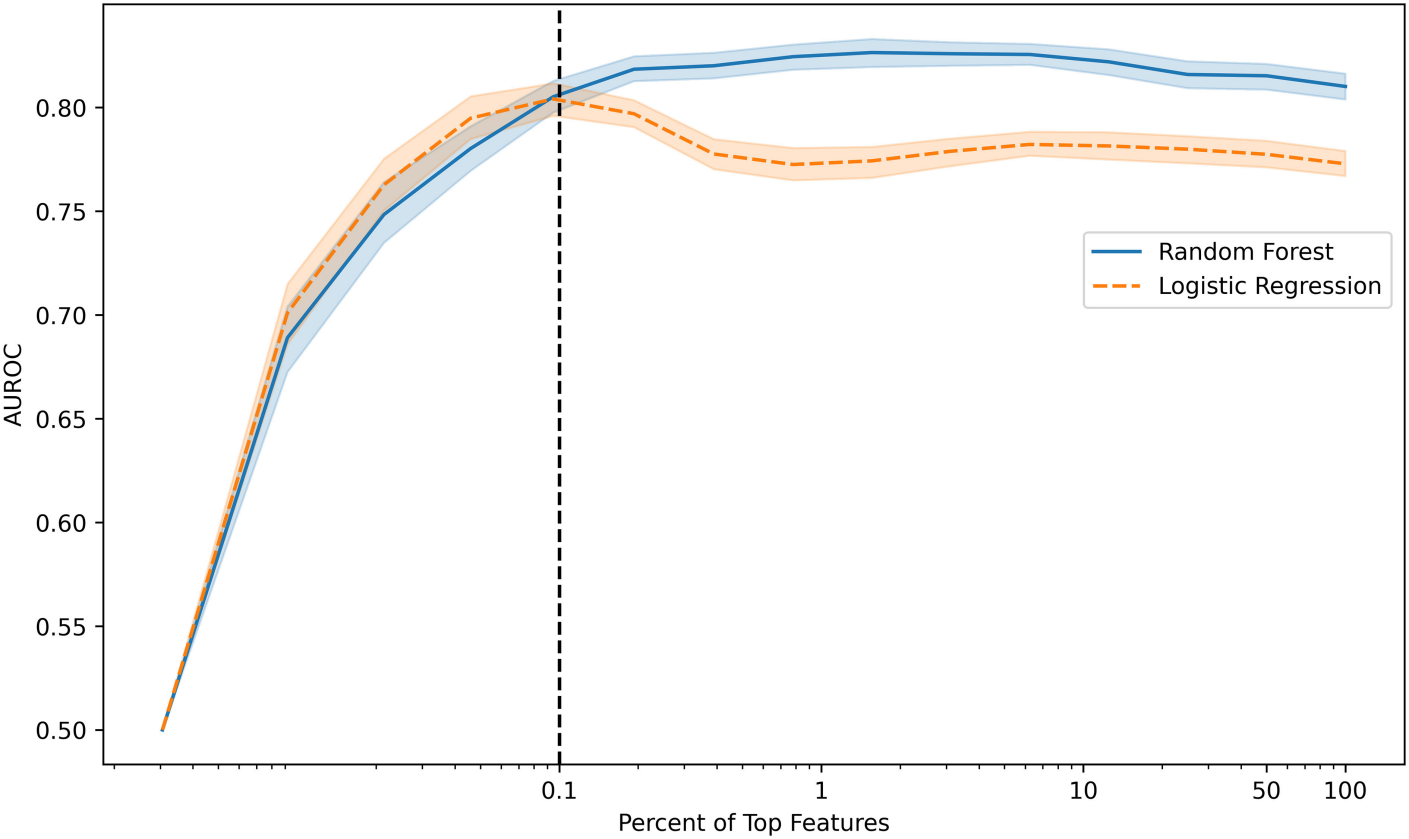
Model performance (area under the receiver operating characteristic curve, AUROC) versus percentage of the top features used to develop random forest and logistic regression models. Models maintain high performance with decreasing percentage of top features included before dropping performance with fewer than 0.1% of features or 31 features (vertical dotted line).

Machine learning models were trained to identify ICI-IA in EHR data on the 89 ICI-IA cases and 2362 machine learning controls. We identified 32,682 EHR codes representing all diagnoses, procedures, labs, and medications, with the temporal modifiers for codes occurring before and after ICI initiation as variables for the models. Trained on the full code set, logistic regression modeling achieved an AUROC of 0.77 (95% Confidence Interval [CI] 0.73-0.82) while random forest modeling achieved an AUROC of 0.81 (95% CI 0.76-0.86) (**Table 3**). Training on decreasing percentages of the top codes derived from the random forest model showed consistently high performance for logistic regression and random forest models before dropping in performance below 31 codes (**Figure 2**). Our highest performing model, random forest with the top 31 codes, achieved an AUROC of 0.81 (95% CI 0.75-86), accuracy of 0.79 (95% CI 0.63-0.94), sensitivity of 0.71 (95% CI 0.54-0.88), specificity of 0.79 (95% CI 0.62-0.95), PPV of 0.13 (95% CI 0.03-0.22), and NPV of 0.99 (95% CI 0.98-0.99) (**Table 3**).

**Table 3:** Logistic Regression and Random Forest machine learning models performance metrics for identifying ICI-induced inflammatory arthritis (ICI-IA).

Full models (N features = 32,682)						
Model	AUROC	Accuracy	Sensitivity	Specificity	PPV	NPV
Logistic Regression	0.77 (0.73-0.82)	0.72 (0.51-0.92)	0.73 (0.49-0.96)	0.71 (0.50-0.93)	0.09 (0.05-0.14)	0.99 (0.98-1.00)
Random Forest	0.81 (0.76-0.86)	0.77 (0.60-0.93)	0.74 (0.56-0.91)	0.77 (0.59-0.94)	0.11 (0.05-0.18)	0.99 (0.98-0.99)

Models trained on top 31 EHR codes						
Model	AUROC	Accuracy	Sensitivity	Specificity	PPV	NPV
Logistic Regression	0.80 (0.75-0.86)	0.80 (0.65-0.95)	0.70 (0.53-0.87)	0.81 (0.64-0.97)	0.13 (0.06-0.20)	0.99 (0.98-0.99)
Random Forest	0.81 (0.75-0.86)	0.79 (0.63-0.94)	0.71 (0.54-0.88)	0.79 (0.62-0.95)	0.13 (0.03-0.22)	0.99 (0.98-0.99)

Accuracy, sensitivity, specificity, PPV, and NPV were calculated optimizing Youden’s J.

AUROC, area under the receiver operating characteristic curve; PPV, positive predictive value; NPV, negative predictive value.

Confidence intervals in parentheses are 95% confidence intervals calculated by bootstrapping model development 100 times.

### 3.3 Key EHR codes in the ICI-induced inflammatory arthritis model were potentially related to other irAEs

**Figure 3:**
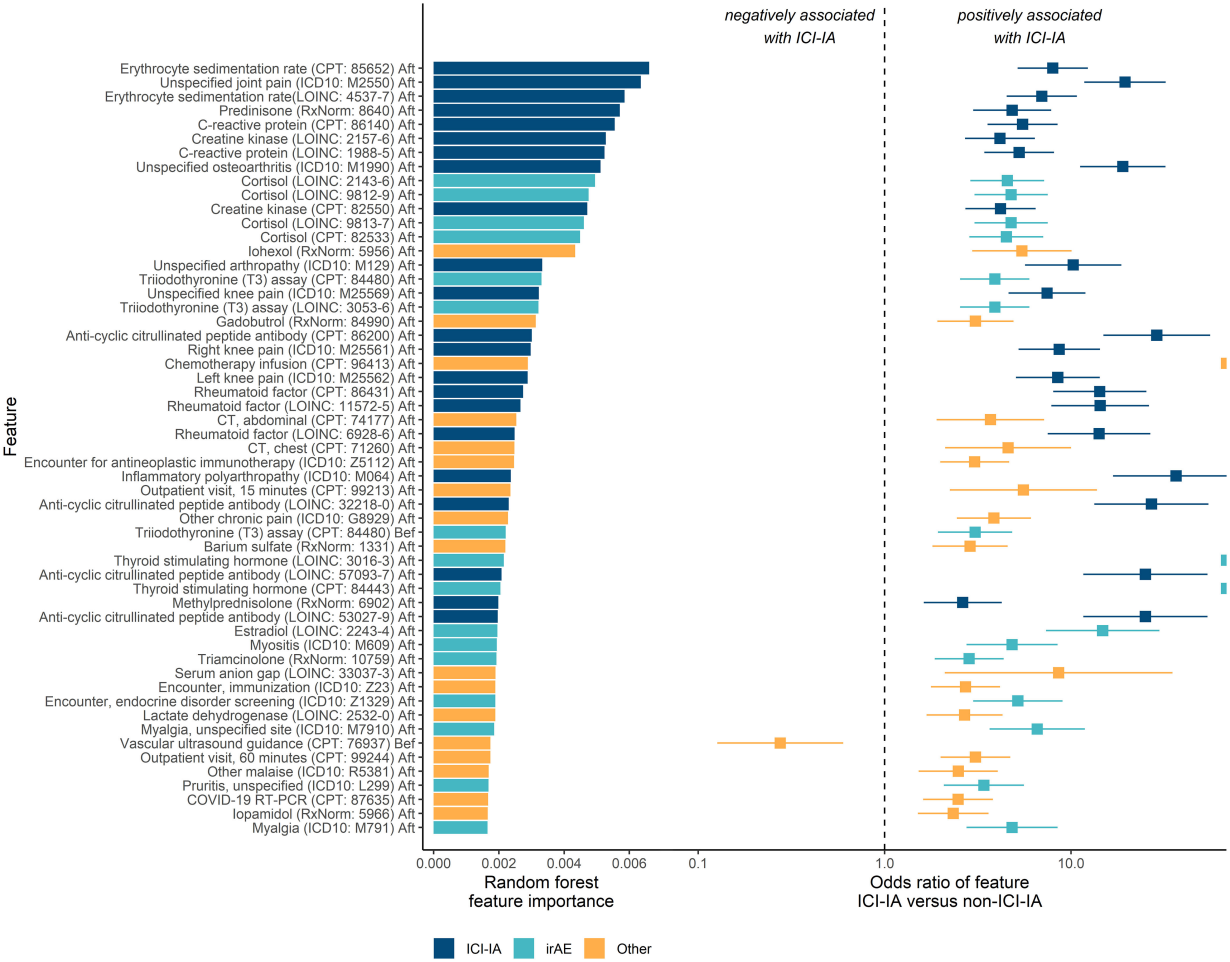
Key EHR codes in the machine learning models and association with ICI-induced inflammatory arthritis (ICI-IA). Left: the random forest model’s feature importance for identifying ICI-IA patients. Right: odds of the patient having an EHR code if they developed ICI-IA versus if they did not, by Fisher Exact test. Error bars are 95% confidence intervals. ‘ICI-IA’ codes are those directly relevant to ICI-IA. ‘irAE’ codes are those potentially describing other irAEs. ‘Other’ codes are those describing other parts of the patient medical history. The top codes are predominantly ICI-IA relevant codes, with a high concentration of relevant codes occupying the topmost importance. The top irAE related codes are endocrine (cortisol, thyroid function tests, estradiol), myositis, and cutaneous (medication order for triamcinolone and pruritus). The majority of the top codes are positively associated with ICI-IA. Codes are labeled by name, code vocabulary, code, and temporal modifier (before or after ICI therapy initiation).

From the random forest model, we evaluated the 55 most important codes to elucidate clinical features relevant to identifying ICI-IA. Codes were categorized as ICI-IA relevant (codes matching elements of ICI-IA presentation, diagnosis, or management in the literature), potentially relevant to other irAEs, and those that were related to other medical history elements. Twenty-two were relevant to ICI-IA, including diagnosis codes for unspecified joint pain, knee pain, and unspecified osteoarthritis (osteoarthritis was likely a placeholder early in the diagnostic workflow); laboratory test and procedure codes for erythrocyte sedimentation rate (ESR), C-reactive protein (CRP), rheumatoid factor, and anti-CCP antibody tests; medication codes for prednisone and methylprednisolone. (‘ICI-IA’ codes in **Figure 3**). Sixteen were potentially relevant to other irAEs, including codes for endocrine disorder screening, thyroid function tests, cortisol labs, medication codes for triamcinolone, and diagnosis codes for pruritus and myositis (‘irAE’ codes in **Figure 3**). Seventeen were related to other medical history elements such as COVID-19 or chemotherapy administration (‘Other’ codes in **Figure 3**).

### 3.4 Melanoma, renal cell carcinoma, and combination nivolumab-ipilimumab therapy were associated with development of ICI-induced inflammatory arthritis

Melanoma, OR = 1.99 (95% CI 1.08-3.65) and renal cell carcinoma patients, OR = 2.03 (95% CI 1.06-3.84), had higher odds of developing ICI-IA compared to lung cancer patients, adjusting for sex, age, race, ethnicity, and first ICI regimen. Combination nivolumab-ipilimumab treatment was associated with higher odds of developing ICI-IA, OR = 1.86 (95% CI 1.01-3.43), compared to patients who received pembrolizumab, adjusting for sex, age, race, ethnicity, and cancer type (**Table 4**).

**Table 4:** Odds of developing ICI-induced inflammatory arthritis (ICI-IA) given cancer and first ICI.

Cancer	Unadjusted OR (95% CI)	P-value^†^	Adjusted OR (95% CI)	P-value^†^
Lung Cancer	1.00 (reference)		1.00 (reference)	
Melanoma	2.28 (1.36-3.78)	**0.001**	1.99 (1.08-3.65)	**0.026**
Renal Cell Carcinoma	2.42 (1.35-4.22)	**0.002**	2.03 (1.06-3.84)	**0.031**
Breast Cancer	1.66 (0.09-8.29)	0.624	1.67 (0.09-8.91)	0.627
Endometrial Cancer	3.05 (0.17-16.16)	0.291	2.21 (0.12-12.33)	0.460
Urothelial Cancer	0.52 (0.08-1.73)	0.371	0.70 (0.11-2.43)	0.638
Other Malignancy	1.33 (0.21-4.53)	0.700	1.25 (0.20-4.40)	0.763

First ICI	Unadjusted OR (95% CI)	P-value^†^	Adjusted OR (95% CI)	P-value^†^
Pembrolizumab	1.00 (reference)		1.00 (reference)	
Nivolumab	1.23 (0.69-2.14)	0.478	0.95 (0.52-1.71)	0.855
Ipilimumab	0.84 (0.20-2.43)	0.782	0.49 (0.11-1.57)	0.282
Combination*	2.89 (1.67-4.99)	**<0.001**	1.86 (1.01-3.43)	**0.046**
Durvalumab	1.15 (0.39-2.78)	0.780	1.22 (0.40-3.07)	0.703
Atezolizumab	0.37 (0.09-1.04)	0.099	0.40 (0.09-1.16)	0.137

Covariates included for adjusted odds ratios (ORs) were sex, age, race, ethnicity, as well as cancer type for ICI ORs and first ICI regimen for cancer type ORs.

*Combination: Nivolumab-Ipilimumab.

^†^P-values < 0.05 significant (bold).

### 3.5 ICI-induced inflammatory arthritis was associated with development of cutaneous, endocrine, and gastrointestinal irAEs

We further explored the key irAE related EHR codes from our ICI-IA machine learning model. The irAEs most commonly documented as co-occurring with ICI-IA were cutaneous irAE: pruritus, rash, and vitiligo (N=38); gastrointestinal irAE: diarrhea and constipation (N=26) and colitis (N=9); endocrine irAE: thyroid dysfunction (N=16), hypophysitis and adrenal insufficiency (N=10), and diabetes (N=1); and hepatic irAE: transaminitis and hepatitis (N=8). Four ICI-IA patients experienced pneumonitis. Pericarditis and encephalitis were documented in one separate patient each. Twenty-one ICI-IA patients had no other documented irAE.

ICI-IA patients had higher odds of having any additional documented irAE, odds ratio (OR) = 2.53 (95% CI 1.49-4.31), compared to ICI-NoArthritis patients, adjusting for sex, age, race, ethnicity, cancer type, first ICI regimen, and presence of autoimmune disease prior to ICI therapy (**Table 5**). They specifically had higher odds of experiencing cutaneous irAE, OR = 2.66 (95% CI 1.63-4.35), endocrine irAE, OR = 2.09 (95% CI 1.15-3.80), and gastrointestinal irAE, OR = 2.88 (95% CI 1.76-4.72) (**Table 5**).

**Table 5:** Odds ratio (OR) of developing irAEs given ICI-induced inflammatory arthritis (ICI-IA).

IrAE anytime	Unadjusted Odds of irAE (95% CI) ICI-IA vs controls	Unadjusted p-value^†^	Adjusted Odds of irAE (95% CI) ICI-IA vs controls	Adjusted p-value^†^
Any IrAE	3.10 (1.87-5.15)	**<0.001**	2.91 (1.74-4.87)	**<0.001**
Cutaneous	3.34 (2.12-5.25)	**<0.001**	3.18 (2.00-5.06)	**<0.001**
Endocrine	3.18 (1.85-5.48)	**<0.001**	2.70 (1.54-4.72)	**<0.001**
Thyroid	3.13 (1.71-5.73)	**<0.001**	2.67 (1.43-4.99)	**0.002**
Hypophysitis/Adrenal Insuff.	4.28 (1.99-9.24)	**<0.001**	3.35 (1.52-7.37)	**0.003**
Diabetes	1.97 (0.23-17.04)	0.539	2.53 (0.27-23.64)	0.415
Gastrointestinal	3.09 (1.95-4.90)	**<0.001**	2.90 (1.81-4.66)	**<0.001**
Diarrhea/Constipation	2.71 (1.65-4.46)	**<0.001**	2.59 (1.55-4.31)	**<0.001**
Colitis	2.47 (1.15-5.28)	**0.020**	2.18 (0.99-4.80)	0.054
Hepatic	2.17 (0.98-4.80)	0.057	1.97 (0.86-4.49)	0.107
Pneumonitis	0.61 (0.22-1.73)	0.356	0.64 (0.22-1.81)	0.397
Cardiac	9.89 (0.61-159.42)	0.106	10.74 (0.62-186.55)	0.103
Encephalitis	1.40 (0.17-11.53)	0.753	1.28 (0.15-10.95)	0.820

IrAE Prior to ICI-IA	Unadjusted Odds of irAE (95% CI) ICI-IA vs controls	Unadjusted p-value^†^	Adjusted Odds of irAE (95% CI) ICI-IA vs controls	Adjusted p-value^†^
Cutaneous	1.65 (1.00-2.72)	**0.048**	1.58 (0.95-2.63)	0.077
Endocrine	1.02 (0.47-2.18)	0.963	0.81 (0.37-1.77)	0.595
Thyroid	0.85 (0.33-2.18)	0.735	0.67 (0.26-1.77)	0.421
Hypophysitis/Adrenal Insuff.	2.45 (0.98-6.13)	0.056	1.81 (0.70-4.65)	0.218
Diabetes	1.97 (0.23-17.04)	0.539	2.53 (0.27-23.64)	0.415
Gastrointestinal	1.76 (1.07-2.90)	**0.027**	1.62 (0.97-2.71)	0.065
Diarrhea/Constipation	1.55 (0.88-2.73)	0.126	1.44 (0.81-2.56)	0.215
Colitis	1.87 (0.81-4.32)	0.142	1.66 (0.70-3.93)	0.251
Hepatic	1.03 (0.36-2.96)	0.954	0.91 (0.31-2.69)	0.863
Pneumonitis*				
Cardiac*				
Encephalitis*				

IrAE Post ICI-IA	Unadjusted Odds of irAE (95% CI) ICI-IA vs controls	Unadjusted p-value^†^	Adjusted Odds of irAE (95% CI) ICI-IA vs controls	Adjusted p-value^†^
Cutaneous	0.91 (0.51-1.62)	0.744	0.82 (0.45-1.48)	0.510
Endocrine	1.76 (0.94-3.32)	0.079	1.45 (0.76-2.79)	0.262
Thyroid	2.01 (1.01-4.00)	**0.045**	1.67 (0.82-3.39)	0.155
Hypophysitis/Adrenal Insuff.	1.59 (0.54-4.68)	0.398	1.24 (0.41-3.69)	0.703
Diabetes*				
Gastrointestinal	0.74 (0.39-1.40)	0.358	0.68 (0.36-1.30)	0.243
Diarrhea/Constipation	0.83 (0.42-1.65)	0.600	0.77 (0.38-1.55)	0.463
Colitis	0.50 (0.12-2.12)	0.351	0.44 (0.10-1.90)	0.271
Hepatic	1.03 (0.36-2.96)	0.954	0.88 (0.30-2.59)	0.815
Pneumonitis	0.61 (0.22-1.73)	0.356	0.64 (0.22-1.81)	0.397
Cardiac	9.89 (0.61-159.44)	0.106	10.74 (0.62-186.55)	0.103
Encephalitis	1.40 (0.17-11.53)	0.753	1.28 (0.15-10.95)	0.820

Covariates included for adjusted odds ratios (ORs) were sex, age, race, ethnicity, cancer type, first ICI regimen, and prior autoimmune disease. IrAE anytime: in cases, irAEs included were those that developed at any time in the patient medical history. IrAE Prior to ICI-IA: in cases, irAEs included were those that developed prior to ICI-IA. IrAE post ICI-IA: irAEs included where those that developed after ICI-IA. In controls, irAEs that developed at any time in the medical history were included for all comparisons. Endocrine irAEs included thyroid, hypophysitis/adrenal insufficiency (Insuff.), and diabetes. Gastrointestinal irAEs included diarrhea/constipation and colitis.

*These irAE had zero cases in our ICI-IA cohort at these timepoints.

^†^P-values < 0.05 significant (bold).

Cases were 89 patients with ICI-IA. Controls were 871 patients without ICI-IA.

In unadjusted models, ICI-IA patients had higher odds of developing cutaneous irAE, OR = 1.65 (95% CI 1.00-2.72), and gastrointestinal irAE, OR = 1.76 (95% CI 1.07-2.90), prior to ICI-IA development compared to the ICI-NoArthritis control developing these irAEs at any time. ICI-IA patients had higher odds of developing thyroid dysfunction, OR = 2.01 (95% CI 1.01-4.00), post ICI-IA development compared to the ICI-NoArthritis control developing thyroid dysfunction at any time (**Table 5**). However, these temporal associations were no longer statistically significant when adjusting for sex, age, race, ethnicity, cancer type, first ICI regimen, and presence of prior autoimmune disease.

## 4 Discussion

Using an ICI cohort of 2451 patients, 89 of which had ICI-IA, we developed an EHR-based machine learning model that could rapidly identify patients who had possible ICI-IA with AUROCs of 0.80-0.81 and accuracy of 0.79-0.80. Our machine learning model captured key EHR codes relevant to ICI-IA as well as codes indicative of cutaneous and endocrine irAEs. On further investigation of these irAE related codes, we found that ICI-IA was associated with development of additional cutaneous, endocrine, and gastrointestinal irAEs independent of cancer type and ICI regimen.

To develop our EHR-based machine learning model of ICI-IA, we created a cohort of 2451 patients who had cancer and received an ICI, including 89 ICI-IA patients, and 871 patients without ICI-IA, who were fully adjudicated for irAEs by manual chart review. We expanded on an approach described by Thangaraj et al. and used all diagnosis, laboratory test, procedure, and medication codes in the EHR to develop an ICI-IA identification algorithm (43). Both logistic regression and random forest machine learning models performed well on the full code set (32,682 codes) and maintained high performance while reducing the number of EHR codes used to develop the models – at 31 codes, logistic regression had an AUROC of 0.80 (95% CI 0.75-0.86) while random forest had an AUROC of 0.81 (95% CI 0.75-0.86) However, both models had low PPVs of 0.13, despite good AUROC and accuracy. This is an inherent limitation of PPV in rare conditions such as ICI-IA, with a prevalence only 3.6% in our ICI cohort. This also speaks to the purpose of our ICI-IA model as a filter for potential ICI-IA cases for clinician verification rather than as a replacement of clinical expertise.

A major advantage of this approach compared to traditional manual selection of model features is that our EHR model provided information on codes useful for identifying ICI-IA patients. While these codes are not a direct equivalence, they were indicators of clinical features that could describe ICI-IA. Both models maintained high performance down to 0.1% of the total code set, suggesting the majority of information important for identifying ICI-IA patients was held by a small fraction of EHR codes. We evaluated the top codes from the random forest model to understand the key predictive elements. Twenty-two of the 55 top codes were determined to be relevant to ICI-IA including lab and procedure codes for ESR, CRP, and rheumatoid factor, diagnosis codes for joint pain, and medication codes for steroids. These factors are consistent with the ICI-IA literature regarding ICI-IA presentation, diagnostic tests, and therapy (27, 29, 37, 49). The density of ICI-IA relevant codes in the group of features with the highest feature importance – the top 8 codes were ICI-IA relevant codes – indicates that our strategy constructed a clinically sensible model to capture ICI-IA relevant information without manual guidance on which codes should be used, increasing the credibility of the other unexpected key EHR codes we found.

While many of the top codes identified by our models were similar to those identified by clinical experts in ICI-IA, 16 of the 55 top codes appeared related to other irAEs, suggesting a possible relationship between ICI-IA and other irAE. We found codes for thyroid stimulating hormone (TSH) and T3 thyroid function tests, cortisol tests, pruritus, and triamcinolone, all after ICI initiation. These codes indicated potential association of ICI-IA with endocrine and cutaneous irAE. Indeed the literature suggests comorbid cutaneous, endocrine, gastrointestinal, and other irAEs with development of ICI-IA (26, 27). However, to our knowledge we are the first to statistically associate that ICI-IA patients were significantly more likely than ICI patients without ICI-IA to also develop cutaneous, OR = 2.66 (95% CI 1.63-4.35), endocrine, OR = 2.09 (95% CI 1.15-3.80), and gastrointestinal irAEs, OR = 2.88 (95% CI 1.76-4.72), adjusting for sex, age, race, ethnicity, cancer type, first ICI regimen, and presence of autoimmune disease prior to ICI initiation. ICI-IA patients were also more likely to experience any other irAE, OR = 2.53 (95% CI 1.49-4.31). To our knowledge, this is the first study that has conducted tests of association of irAEs comparing ICI-IA to a control cohort of ICI patients without ICI-IA, with both cohorts fully adjudicated for irAEs. This confirms the results of previous studies (26, 30, 32, 38) and shows that patients with ICI-IA are actually experiencing higher rates of cutaneous, endocrine, and gastrointestinal irAE compared to the general ICI population. Additionally, our findings suggest that the association between ICI-IA and cutaneous, endocrine, and gastrointestinal irAEs is independent of cancer type, ICI regimen, and prior autoimmune disease.

Conducting temporal association tests for irAEs that occurred before and after ICI-IA, we found that ICI-IA patients were more likely to have developed cutaneous irAEs and gastrointestinal irAEs prior to ICI-IA, in an unadjusted model. Conversely, ICI-IA patients were more likely to develop thyroid dysfunction after ICI-IA. Our temporal association findings indicate a possible common phenotype of patients who receive ICI therapy, develop skin and/or gastrointestinal irAEs, followed by ICI-IA, and finally thyroid dysfunction. However, when adjusting for demographics, cancer type, ICI regimen, and prior autoimmune disease, these temporal associations were no longer statistically significant, suggesting that the temporal phenotypes may be dependent on cancer type and ICI regimen or specific to our cohort and will require larger cohort sizes and future study.

As part of our analysis, we recapitulated findings in the literature of associations between cancer and ICI regimen and development of ICI-IA. We found similar associations in our ICI-IA cohort to that observed previously by Cunningham-Bussel et al. (38) even with our differing inclusion protocol for ICI-IA, increasing our confidence in the representativeness of our ICI-IA and ICI cohorts. Melanoma patients, OR = 1.99 (95% CI 1.08-3.65), and renal cell carcinoma patients, OR = 2.03 (95% CI 1.06-3.84), had higher odds of developing ICI-IA compared to lung cancer patients, adjusting for sex, age, race, ethnicity, and first ICI regimen. We also found that patients whose first ICI regimen was combination nivolumab-ipilimumab had higher odds of developing ICI-IA, OR = 1.86 (95% CI 1.01-3.43), compared to patients who received pembrolizumab, adjusting for sex, age, race, ethnicity, and cancer type. We additionally found similar time elapsed from ICI initiation to ICI-IA development compared to the ICI-IA literature – median of 20 weeks (5 months) compared to 2.7 – 5 months (30, 34, 50).

Our study is not without limitations. Despite being larger than most published studies, our ICI-IA cohort is still relatively small (N=89) and constrained to a single site. This small cohort relative to the number of EHR codes could make training our machine learning models difficult. We mitigated this issue by performing feature reduction based on the feature importance provided by the initial random forest model and saw high-performance using only 0.1% of the total code set. Another limitation is that our ICI-IA adjudication was done by retrospective chart review, therefore relying on clinician documentation of ICI-IA, and included patients that were not seen by a rheumatologist. Thus, some of these patients may have arthralgia or polymyalgia rheumatica rather than arthritis. However, as we expand this study to further sites and patients, we will be able to better make this distinction. We chose to accept these limitations to capture a broader cohort of patients with potentially less severe ICI-IA or who had other barriers to seeing a rheumatologist. Our finding that only 18 of the 89 ICI-IA patients were seen by a rheumatologist further highlights the shortcomings of the current standard for defining ICI-IA cohorts and the importance of our model for identifying ICI-IA patients.

In summary, we developed a novel EHR-based machine learning algorithm that was able to identify ICI-IA patients in the EHR with high performance. This EHR algorithm could be adapted to other EHR systems to facilitate cohort definition for ICI-IA for multicenter research studies and to assist in clinical practice with recommending patients suspected to have ICI-IA to follow-up care with a rheumatologist. Our machine learning strategy revealed hidden relationships between ICI-IA and other irAE that were corroborated in association tests. It provided insight into the clinical features that were descriptive of ICI-IA, including features potentially pertaining to comorbid irAEs. By further examining these irAEs, we found that cutaneous, endocrine, and gastrointestinal irAEs were significantly more likely to be found in patients with ICI-IA compared to cancer patients receiving ICI therapy who did not experience ICI-IA, independent of cancer type and ICI regimen. We also found potential temporal relationships between ICI-IA and other irAEs. This indicates that other such temporal phenotypes may exist in patients who develop irAEs that could be captured in EHR data. Overall, further exploration of irAEs in EHR data and investigation of irAE temporal phenotypes may reveal leads for mechanistic studies of irAE development and improve care for these patients through more rapid identification of irAEs and timelier recommendation for follow-up care.

## Data availability statement

The data analyzed in this study is subject to the following licenses/restrictions: The data used and analyzed during the current study are available from the corresponding author on reasonable request. Note that row level data is access controlled and cannot be provided publicly due to data use restrictions. Models are available on GitHub (https://github.com/stevetran99/ICI-IA_ML_Classification). Requests to access these datasets should be directed to TLW, t-walunas@northwestern.edu.

## Ethics statement

The studies involving humans were approved by Northwestern University Institutional Review Board. The studies were conducted in accordance with the local legislation and institutional requirements. The ethics committee/institutional review board waived the requirement of written informed consent for participation from the participants or the participants’ legal guardians/next of kin because impracticality of consent and low risk of the study.

## Author contributions

SDT: Data curation, Formal Analysis, Funding acquisition, Investigation, Methodology, Project administration, Resources, Software, Visualization, Writing – original draft, Writing – review & editing, Conceptualization. JL: Data curation, Writing – review & editing, Methodology. CG: Data curation, Methodology, Writing – review & editing. LVR: Data curation, Methodology, Writing – review & editing. JP: Data curation, Methodology, Writing – review & editing. GMP: Data curation, Writing – review & editing. KJR: Data curation, Writing – review & editing. CDM: Data curation, Writing – review & editing. JDJ: Data curation, Writing – review & editing. JT: Data curation, Writing – review & editing. KV: Data curation, Writing – review & editing. PVD: Data curation, Writing – review & editing. SM: Data curation, Writing – review & editing. UR: Data curation, Writing – review & editing. KT: Data curation, Writing – review & editing. NM: Investigation, Writing – review & editing. JLJ: Investigation, Writing – review & editing. YL: Investigation, Methodology, Writing – review & editing. AK: Investigation, Writing – review & editing. JS: Investigation, Writing – review & editing. TLW: Writing – review & editing, Conceptualization, Funding acquisition, Investigation, Methodology, Project administration, Resources, Supervision.
