# Supplementary material for: Rapid identification of inflammatory arthritis and associated adverse events following immune checkpoint therapy: a machine learning approach
Source: Front Immunol. 2024 Mar 15;15:1331959. doi: 10.3389/fimmu.2024.1331959 (PMC10978703; doi:10.3389/fimmu.2024.1331959)
Supplement: Supplementary file 1 [file Table_1.docx]

Supplementary Material

# Supplementary Reference Tables

| **Supplemental Table 1.** Cancer and ICI electronic health record data definitions | |
| --- | --- |
| **Cancer/Immunotherapy** | **Codes** |
| Melanoma | ICD-9-CM: 172 ICD-10-CM: C43 |
| Renal cell carcinoma | ICD-9-CM: 189 ICD-10-CM: C64 |
| Lung cancer | ICD-9-CM: 162 ICD-10-CM: C34 |
| Urothelial cancer | ICD-9-CM: 188 ICD-10-CM: C67 |
| Head and neck cancer | ICD-9-CM: 140, 141, 142, 143, 144, 145, 146, 147, 148, 149, 160, 161 ICD-10-CM: C00, C01, C02, C03, C04, C05, C06, C07, C08, C09, C10, C11, C12, C13, C14 |
| Gastric cancer | ICD-9-CM: 151 ICD-10-CM: C16 |
| Colon cancer | ICD-9-CM: 153 ICD-10-CM: C18 |
| Liver cancer | ICD-9-CM: 155 ICD-10-CM: C22 |
| Cervical cancer | ICD-9-CM: 180 ICD-10-CM: C53 |
| Endometrial cancer | ICD-9-CM: 179, 182 ICD-10-CM: C54, C55 |
| Breast cancer | ICD-9-CM: 174, 175 ICD-10-CM: C50 |
| Hodgkins disease | ICD-9-CM: 201 ICD-10-CM: C81 |
| Merkel cell carcinoma | ICD-9-CM: ‘209.31’, ‘209.32’, ‘209.33’, ‘209.34’, ‘209.35’, ‘209.36’ ICD-10-CM: C4A |
| Rectal cancer | ICD-9-CM: 154.1 ICD-10-CM: C20 |
| Prostate cancer | ICD-9-CM: 185 ICD-10-CM: C61 |
| Esophageal cancer | ICD-9-CM: 150 ICD-10-CM: C15 |
| Leukemia | ICD-9-CM: 204, 205, 206, 207, 208 ICD-10-CM: C91, C92, C93, C94, C95 |
| Other lymphoma | ICD-9-CM: 200, 202 ICD-10-CM: C82, C83, C84, C85, C86, C88 |
| Other cancer | ICD-9-CM: 195 ICD-10-CM: C76 |
| Pembrolizumab | Regex: 'pembrolizumab', 'keytruda' |
| Nivolumab | Regex: 'nivolumab', 'opdivo' |
| Cemiplimab | Regex: 'cemiplimab', 'libtayo' |
| Avelumab | Regex: 'avelumab', 'bavencio' |
| Durvalumab | Regex: 'durvalumab', 'imfinzi' |
| Atezolizumab | Regex: 'atezolizumab', 'tecentriq' |
| Ipilimumab | Regex: 'ipilumumab', 'yervoy' |
| Tremilumumab | Regex: 'tremilumumab' |

| **Supplemental Table 2.** Autoimmune disease electronic health record definitions | |
| --- | --- |
| **Autoimmune Disease** | **Codes** |
| Endocrine: Hashimoto's thyroiditis | ICD-9-CM: 245  ICD-10-CM: E06.3 |
| Endocrine: T1D | ICD-9-CM: 250.01, 250.03, 250.11, 250.13, 250.21, 250.23, 250.31, 250.33, 250.41, 250.43, 250.51, 250.53, 250.61, 250.63, 250.71, 250.73, 250.81, 250.83, 250.91, 250.93  ICD-10-CM: E10 |
| GI: Autoimmune hepatitis | ICD-9-CM: 571.42  ICD-10-CM: K75.4 |
| GI: Crohn's disease | ICD-10-CM: K50 |
| GI: Primary biliary cholangitis (PBC) | ICD-10-CM: K74.3, K74.5 |
| GI: Ulcerative colitis (UC) | ICD-9-CM: 556  ICD-10-CM: K51 |
| Neuro: Guillain-Barre Syndrome | ICD-10-CM: G61.0 |
| Neuro: Lambert-Eaton syndrome | ICD-9-CM: 358.3  ICD-10-CM: G70.8, G73.1 |
| Neuro: Multiple sclerosis | ICD-9-CM: 340  ICD-10-CM: G35 |
| Neuro: Myasthenia gravis | ICD-9-CM: 358.0, 775.2  ICD-10-CM: G70.0 |
| Neuro: Myelitis transversa | ICD-9-CM: 341.2  ICD-10-CM: G37.3 |
| Skin: Dermatitis herpetiformis | ICD-9-CM: 694.0, 694.2  ICD-10-CM: L13.0 |
| Skin: Pemphigoid | ICD-9-CM: 694.5, 694.6  ICD-10-CM: L12 |
| Skin: Pemphigus | ICD-9-CM: 694.4  ICD-10-CM: L10 |
| Skin: Psoriasis | ICD-9-CM: 696.1, 696.2, 696.8  ICD-10-CM: L40, L41 |
| Skin: Pyoderma | ICD-9-CM: 686.0  ICD-10-CM: L08.0, L88 |
| Skin: Vitiligo | ICD-9-CM: 709.1  ICD-10-CM: L80, H02.73 |

# Supplementary Methods

## Manual chart review process

Clinical notes were filtered for history and physical examination (H&P) and progress notes occurring on or after immune checkpoint inhibitor (ICI) therapy initiation. All H&P and progress notes were reviewed looking for the indicated immune-related adverse events (irAE), focusing on the assessment and plan sections. Common keywords for each irAE are listed. IrAE was classified if the oncologist or relevant specialist noted suspicion that the presentation was secondary to ICI therapy. This chart review pipeline was developed by SDT with guidance by CG, JL, AK, and JS. SDT trained GMP, KJR, CDM, JDJ, JT, KV, PD, SM, UR for chart review with this guide. SDT additionally reviewed 50 random charts from the other reviewers to compare agreement of the irAEs found – this resulted in greater than 90% agreement.

| **Supplemental Table 3.** IrAE chart review clinical note specialties and keywords. | | |
| --- | --- | --- |
| irAE | Specialty | Keywords |
| Cutaneous | Oncology, Dermatology | rash, pruritis, Vitiligo, Stevens-Johnson syndrome (SJS), Toxic epidermal necrolysis (TEN) |
| Thyroid dysfunction | Oncology, Endocrinology | abnormal TSH, thyroiditis, hypothyroidism, thyroid dysfunction |
| Hypophysitis/adrenal insufficiency | Oncology, Endocrinology | hypophysitis, adrenal insufficiency, abnormal ACTH, abnormal cortisol |
| Diabetes | Oncology, Endocrinology | diabetes, ketoacidosis, abnormal A1C, abnormal glucose |
| Diarrhea/Constipation | Oncology, Gastroenterology | diarrhea, constipation, abnormal bowel movements |
| Colitis | Oncology, Gastroenterology | colitis, GI related ED visit, abnormal bowel movements |
| Hepatic | Oncology, Hepatology | hepatitis, cholangitis, transaminitis |
| Pneumonitis | Oncology, Pulmonology | pneumonitis, pneumonia (confirm if this is referring to pneumonitis), pulmonary related ED visit |
| Cardiac | Oncology, Cardiology | myocarditis, pericarditis, myopericarditis |
| Encephalitis | Oncology, Neurology | encephalitis |
